# World Health Organization Global Conference on Severe Acute Respiratory Syndrome

**DOI:** 10.3201/eid0909.030559

**Published:** 2003-09

**Authors:** David Bell, Philip Jenkins, Julie Hall

**Affiliations:** * World Health Organization, Geneva, Switzerland

On June 17–18, 2003, in Kuala Lumpur, Malaysia, the World Health Organization (WHO) sponsored a conference entitled SARS: Where Do We Go From Here? The purpose of the conference, which was attended by over 900 scientific and public health experts from 43 countries, was to review available knowledge and lessons learned and to identify key priorities for the future. Three overarching questions were addressed: Can severe acute respiratory syndrome (SARS) be eradicated? Are current control measures effective? Are current alert and response systems robust enough?

The first day included summaries of the history of the epidemic, global, and regional responses coordinated by WHO through its headquarters in Geneva and the Western Pacific Regional Office in Manila, respectively; and national responses in the People’s Republic of China (PRC), including in the Hong Kong Special Administrative Region of PRC, Singapore, Vietnam, Canada, and United States. Nine presentations summarized scientific, clinical, public health, psychosocial, and communications aspects of the SARS outbreak. On the second day, breakout groups met and presented recommendations on the topics of epidemiology and public health, possible role of animals, environmental issues, modeling the epidemic, clinical diagnosis and management, reducing transmission in healthcare settings, blood safety, reducing community transmission, preventing international spread, surveillance and response coordination, effective communication, and preparedness. Background materials for the conference, slide presentations at the plenary sessions (including the breakout group reports), and the text of speeches by the Director General of WHO and other dignitaries are available on the Web (URL: www.who.int/csr/sars/conference).

Beginning in March 2003, after WHO recognized, through its Global Outbreak Alert and Response Network (GOARN), an outbreak of severe respiratory illness with high transmissibility in healthcare settings and international spread through airline travel, WHO issued a series of global alerts, travel advisories, and recommendations for diagnosis, clinical management, and prevention of transmission. Evolving information was discussed by virtual networks of experts, including virologists, clinicians, and epidemiologists. Field teams composed of staff from GOARN partners were quickly mobilized to assist affected countries in enhancing surveillance and containment measures, which included isolating cases, implementing strict infection control measures, identifying and following-up with contacts, and making recommendations to travelers to prevent international spread.

From a global perspective, the SARS epidemic demonstrated the importance of a worldwide surveillance and response capacity to address emerging microbial threats through timely reporting, rapid communication, and evidence-based action. The importance of international collaboration coordinated by WHO and the need for partnerships among clinical, laboratory, public health, and veterinary communities were emphasized. From the national perspective, lessons learned included the need for the following: strong political leadership at the highest levels to mobilize the entire society; speed of action; improved coordination between national and district levels in countries with federal systems; increased investment in public health; updated legislation regarding surveillance, isolation, and quarantine measures; and improved infection control in healthcare and long-term-care facilities and at borders.

## Can SARS Be Eradicated?

The breakout groups concluded that it is too soon to tell if SARS can be eradicated, but substantial reasons for concern exist. Chains of person-to-person transmission can likely be terminated, provided no reservoir of asymptomatic carriers, chronic infection, or seeding of new settings (e.g., Africa) exists. But if an animal reservoir of the SARS coronavirus exists, as suggested by some studies, eradication would be very difficult. Fecal shedding of virus by infected persons and apparent virus stability in the environment could pose additional barriers to eradication, although these circumstances were not major modes of transmission in the recent epidemic. Research priorities include better understanding of the epidemiologic and virologic parameters of infection and transmission, including “superspreading events”; the possible role of animals, including host range and factors leading to emergence; the environment; and analysis of the effectiveness of specific interventions in controlling the epidemic. Additional priorities include standardization of diagnostic assays and reagents, development of a reliable front-line diagnostic test for use early in illness; facilitating the ability to ship diagnostic specimens; and development of animal models to improve understanding of pathogenesis and evolution of clinical disease and to use in vaccine development and antiviral drug testing.

## Are Current Control Measures Effective?

Currently recommended measures to prevent transmission in healthcare settings are generally effective when applied, but require proper infrastructure, training, and consistent practice. Infection control capacity and practice in many healthcare settings need improvement. A minimum global level of safe practice (standard precautions, supplemented by risk-based precautions) should be established. Studies are needed to determine optimal protective measures (e.g., type of mask) and when they should be used. Appropriate protective measures (e.g., isolation facilities and masks fit-tested for individual workers) should be more widely available.

Measures to control community transmission (i.e., outside of healthcare settings) and prevent international spread require further evaluation. Such measures include public information campaigns, contact tracing and sometimes quarantine, hotlines to report fever, temperature screening in public places, recommendations to travelers, and entry and exit screening at borders with questionnaires and temperature checks. Control measures in the community would likely have the greatest yield if focused on links between healthcare settings and the wider community, with contact tracing prioritized according to the nature of exposure, but further evaluation is needed. Home or institutional quarantines, when used, should ensure financial and psychosocial support and daily needs of the affected persons. Stigmatization of affected persons and groups was identified as an important issue. In an attempt to reduce stigmatization, one country’s president reportedly proclaimed quarantined persons to be “heroes in the nation’s battle against SARS.” Some participants stated that visible measures to control community and international spread were important in restoring public and business confidence and as deterrents, regardless of the yield of SARS cases detected.

## Are Current Alert and Response Systems Robust Enough?

Current systems are robust in that SARS is being controlled, but many processes are not sustainable because of limited capacity. Surveillance priorities include developing a sensitive “alert” case definition in areas at greatest risk for recurrence, developing a front-line laboratory diagnostic test to identify patients with SARS coronavirus infection during periods of high incidence of other respiratory illnesses, improving laboratory diagnostic capacity and laboratory-based surveillance, and developing integrated information tools that allow real time analysis of clinical, epidemiologic, and laboratory data.

Response coordination priorities include development of contingency plans, including ensuring coordination and surge capacity at global, regional, and national levels; development of laboratory and information technology systems; and the ongoing revision of the international health regulations to focus on containing emerging infectious diseases.

Underlying any response is the need to communicate information in a transparent, accurate, and timely manner. Effective communication requires training, understanding, and use of a range of different media. Developing further the current communication systems and our understanding of risk communication is vital if future outbreaks are to be controlled quickly and effectively and the health, economic, and psychosocial effects of major health events are to be minimized.

